# The Particle Size of Wheat Bran Dietary Fiber Influences Its Improvement Effects on Constipation

**DOI:** 10.3390/foods14061001

**Published:** 2025-03-15

**Authors:** Luyao Li, Linlin Hu, Rui Chen, Ruoyan Yang, Lingxiao Gong, Jing Wang

**Affiliations:** 1 Key Laboratory of Geriatric Nutrition and Health, Beijing Technology and Business University (BTBU), Ministry of Education, Beijing 100048, China; liluyao13053686838@163.com (L.L.); 17806022328@163.com (L.H.); 2330201019@st.btbu.edu.cn (R.C.); ruoyan127@163.com (R.Y.); 2 School of Food and Health, Beijing Technology and Business University (BTBU), Beijing 100048, China; 3 National Center of Technology Innovation for Grain Industry, Comprehensive Utilization of Edible by-Products, School of Food and Health, Beijing Technology and Business University (BTBU), Beijing 100048, China

**Keywords:** dietary fiber, particle size, constipation, gut microbiota

## Abstract

Wheat bran dietary fiber (WBDF) is a potential functional additive to enrich products used for relieving constipation. The purpose of this study was to understand the effects of different particle size ranges (mean sizes of 84.14, 61.74, 37.39, and 22.33 μm) of WBDF on constipation. With the decrease in particle size, its morphology exhibited an increase in fiber fragmentation, larger pore sizes, and the formation of structural faults. The oil-holding capacity (OHC) and swelling capacity (SC) of WBDF were found to change with particle size, with the highest OHC observed at 37.39 μm and the greatest SC at 84.14 μm. Animal experiments demonstrated that the WBDF of smaller particle sizes significantly alleviated loperamide-induced constipation with an increased intestinal propulsion rate, decreased first melanin excretion time, and reduced gastric residual rate. Meanwhile, WBDF samples markedly increased serum MTL and serum AChE levels. Notably, compared with the constipation model (CMNC) group, the small intestinal propulsion rate in the MPS40 group increased by 41.21%, and the gastric residue rate significantly decreased by 19.69%. The improvement in constipation symptoms was most pronounced. Additionally, the abundance of *Lactobacillus* in the MPS40 group increased by 52.52%, while the relative abundance of *Prevotella* decreased by 83.55%, and the diversity of the gut microbiota was altered. These findings provide valuable insights into the potential commercial applications of WBDF in fiber-enriched functional foods to support intestinal health.

## 1. Introduction

Constipation is a prevalent functional gastrointestinal disorder worldwide. Clinical symptoms include low stool volume, dry stools, and difficulty in defecation, and they are accompanied by bloating and loss of appetite. The common global occurrence of constipation is about 11–20%, with a higher incidence in adults (around 16%) compared to teenagers (about 9.5%) [1,2,3]. Gastrointestinal hormones are a group of peptides distributed in the gastrointestinal tract and bloodstream, playing an important role in the regulation of gastric motility [4]. The management and therapy of constipation can be achieved not only with drugs but also with nonpharmacologic management such as a high-fiber diet [5,6]. Dietary fiber (DF) could not only alleviate constipation but could also prevent it in the first place. Although this is well known, constipation remains prevalent due to differences in individual dietary habits and metabolic genetic factors.

Dietary fiber (DF), as defined by the World Health Organization and Codex Alimentarius, encompasses carbohydrates that are not digested or absorbed in the small intestine. These fibers typically have a polymerization degree (DP) of ten or more monomeric units and are categorized into soluble and insoluble dietary fiber (SDF and IDF) based on their solubility. DF primarily includes non-starch polysaccharides such as cellulose, hemicelluloses, pectins, resistant starch, and certain non-digestible oligosaccharides. The recommended daily intake of dietary fiber for adults is usually between 25 and 35 g; however, the average intake tends to be under 20 g [7,8]. DF possesses high nutritional value and numerous physiological functions, playing a key role in alleviating constipation, maintaining healthy body weight, reducing the risk of coronary heart disease, lowering blood lipid and blood glucose levels, and exhibiting anti-gastrointestinal cancer properties [9]. The effect of DF on constipation is influenced by the physicochemical properties of fibers, as well as the dose and duration of the intervention [10]. These varying factors result in differences in the water-holding capacity of DF, its transit time in the gastrointestinal tract, and the stimulation of intestinal bacterial proliferation. Peng et al. observed that varying doses of the insoluble dietary fiber extract from wild apricots resulted in an enhanced small intestine transit rate in mice, as well as a reduction in the time taken for the first defecation [11]. Additionally, the particle size of DF is an important characteristic that indirectly influences gastrointestinal physiological functions, including gastrointestinal hormones, transit time, and fecal excretion [12]. Previous research by Jenkins et al. indicated that smaller WBDF particles increased butyrate levels, suggesting potential benefits for colonic mucosal health [13]. A previous study investigated how the particle size of ultrafine asparagus pomace fiber powder influences gut characteristics and overall health. The results demonstrated that fiber of a smaller size showed better effects in improving intestinal propulsion rate, reducing the black stool time and increasing defecation quality in the loperamide hydrochloride-induced constipation mice compared to fiber with larger particle sizes [14]. The current conclusion suggests that smaller WBDF particles improve intestinal motility, microbiota composition, and gut hormone levels, indicating multifaceted benefits beyond colonic bulk formation. As a result, exploring the connection between the physical structure and physicochemical characteristics of dietary fiber with varying particle size distributions, along with their effects on gastrointestinal motility and hormone levels following ingestion, is of considerable importance.

Wheat (*Triticum aestivum*) bran is a product of wheat processing, widely available and cost-effective. As a key ingredient in whole-grain foods, it plays an important role in health functions and offers high nutritional value. Wheat bran, a prominent source of DF, is recommended for relief of constipation due to its outstanding viscosity, fermentability, and affordability [15,16]. Appropriate intake of DF can effectively modulate the gut microbiota associated with constipation, such as increasing the abundance of beneficial bacteria, including *Bacteroidetes*, *Ruminococcus*, and *Akkermansia*, thereby alleviating constipation [17,18]. Inadequate intake of DF may lead to reduced microbial diversity and depletion of specific bacterial groups in the gut [19,20]. However, the relationship between the particle size of DF and constipation improvement effects is still unknown for wheat bran DF, and further investigation into its health benefits is warranted.

Therefore, our research explores the relationship between the physical structure and physicochemical properties of WBDF with different particle sizes and investigates its multifaceted benefits on intestinal motility, microbiota composition, and gut hormone levels. It also identifies the moderate inclusion of small-particle dietary fiber in the diet as a potential therapeutic approach to alleviate gastrointestinal symptoms in patients with constipation.

## 2. Materials and Methods

### 2.1. Materials

Wheat bran was produced in Xinxiang City, Henan Province, China. Enzyme preparations were from Sigma Chemical Co., Ltd. (Louis, MO, USA). And all other chemical reagents were purchased from China Pharmaceutical and Chemical Reagents Co., Ltd. (Beijing, China). ELISA kits motilin (MTL, MM-0492M1), gastrin (Gas, MM-44405M1), somatostatin (SS, MM-0493M1), and acetylcholinesterase (AChE, MM-0674M1) were obtained from Meimian Industrial Co., Ltd., Yancheng, China.

### 2.2. WBDF Preparation

The wheat bran was treated using four different methods: disintegrator, colloid mill (50 Hz, 30 min), ultra-high pressure homogenizer (40–200 Mpa, 30 min), and ball mill (600 rpm, 30 min).

The wheat bran was soaked in water for 30 min and washed until the washing solution no longer appeared milky white, in order to remove surface starch. The wet bran was collected and dried (drying oven DHG-9030A, Shanghai Yiheng Scientific Instrument Co., Ltd., Shanghai, China). WBDF was extracted based on previous methods with minor modifications [21]. Briefly, 100 g of dried wheat bran was added to distilled water at a ratio of 1:10 (g/mL), and the pH was adjusted to 5.5 using HCl. The mixture was stirred and decomposed in a 55 °C water bath for 4 h (Water bath pot PT 2500 E, Jerell Electric Appliance Ltd., Changzhou, China). Afterwards, 4% α-amylase was added, and the mixture was hydrolyzed at a temperature of 60 °C and pH 6.0 for 45 min (hydrolyzing starch into smaller sugars), followed by boiling at 100 °C for 10 min to inactivate the enzymes. After cooling, 3.6% neutral protease was added, and the mixture was hydrolyzed at a temperature of 45 °C and pH 7.5 for 45 min (breaking down proteins into peptides or amino acids), followed by boiling at 100 °C for 10 min to inactivate the enzymes. After cooling, 1% glycosylase was added, and the mixture was hydrolyzed at a temperature of 60 °C and pH 4.5 for 30 min (breaking down starch into glucose), followed by boiling at 100 °C for 10 min to inactivate the enzymes. After cooling, 3% H_2_O_2_ (*w*/*v*) was added and stirred at ambient temperature for three hours for decolorization, followed by centrifugation (CR22N, Hitachi Ltd., Tokyo, Japan) to obtain the supernatant and precipitate. The precipitate was dried in an oven at 60 °C, and anhydrous ethanol was added to the supernatant at a volume ratio of 1:4. After standing for 12 h for ethanol precipitation, and 3000× *g* centrifugation for 10 min to obtain the precipitate sequentially, the precipitate was dried in an oven at 60 °C. All precipitates were collected as DF.

### 2.3. Physicochemical Properties of WBDF

#### 2.3.1. Particle Size Distribution

The particle size distribution (1–1000 μm) of untreated and treated WBDF was analyzed using a laser particle size analyzer (SALD-MS23, Shimadzu Corporation, Kyoto, Japan). Each particle size was determined using deionized water as a dispersant, and each measurement was repeated three times.

#### 2.3.2. Fourier Transform Infrared Spectroscopy (FT-IR)

Approximately 0.10 g of dry KBr powder and 1 mg of the sample were separately placed in an agate mortar and ground thoroughly. The mixture was then subjected to infrared light analysis using the FTIR spectrophotometer (Thermo Nicolet, Waltham, MA, USA). After grinding, it was evenly added to the mold pressing machine and pressurized to make a thin transparent sheet with a certain diameter, which was immediately scanned in the optical path [22].

#### 2.3.3. Scanning Electron Microscope (SEM)

WBDF samples were characterized using SEM (Carl Zeiss Jena 55, Jena, Germany) [23]. In brief, the sample was held in place by conductive tape and coated with a thin layer of gold. SEM images were then captured at magnifications of 500× and 5000×, using an acceleration voltage of 3.00 kV.

#### 2.3.4. Water-Holding Capacity (WHC)

WHC was determined according to the method described by Lu et al. [24]. A total of 0.50 g (M_0_) WBDF was weighed and mixed with 30 mL of distilled water. The mixture was stirred for three hours. After centrifugation, the supernatant was discarded, and the precipitate was collected and weighed as M_w_. The WHC of WBDF is calculated by Equation (1).

(1) $$WHC\;(g/g)=\frac{M_{W}-M_{0}}{M_{0}}$$

where M_0_ (g) represents the mass of the WBDF prior to mixing, and M_W_ (g) represents the mass of the precipitate following mixing.

#### 2.3.5. Oil-Holding Capacity (OHC)

OHC was determined according to the method described by Lu et al. [24]. A total of 0.50 g (M_0_) WBDF was taken, together with 30 mL peanut oil, and was stirred and mixed for three hours. After centrifugation, the upper oil was carefully discarded, and the precipitate was collected and weighed as M_o_. The OHC of WBDF is calculated by Equation (2).

(2) $$OHC\;(g/g)=\frac{M_{O}-M_{0}}{M_{0}}$$

where M_0_ (g) is the mass of WBDF before mixed, and Mo (g) is the mass of the precipitate after mixed.

#### 2.3.6. Swelling Capacity (SC)

SC was determined according to the method described by Zhang et al. [25]. A total of 0.50 g (M_0_) WBDF was placed into a 10 mL measuring cylinder. After recording the volume V_1_ of WBDF, 8 mL of deionized water was added. The volume V_2_ was recorded after 24 h at room temperature. The SC of WBDF is calculated by Equation (3).

(3) $$SC\;(mL/g)=\frac{V_{2}{-V}_{1}}{M_{0}}$$

where M_0_ (g) is the mass of WBDF, and V_1_ (mL) and V_2_ (mL) are the volume of WBDF before and after adding water.

### 2.4. Animal Experiments

Male Kunming (*Mus musculus* KM) mice (6–8 weeks old, 18–22 g) were obtained from Beijing Vital River Laboratory Animal Technology Co., Ltd. (Beijing, China). After one week of feeding and adaptation, 48 mice were stochastically classified into six groups (*n* = 8): normal control group (NC), loperamide induced constipation model group (CMNC), and experimental groups (MPS90, MPS60, MPS40, and MPS20). Except for the NC group, the remaining groups were intragastrically administered with loperamide hydrochloride (Yangsen Pharmaceutical Co., Ltd., H10910065, Xi’an, China) [26]. The mice in MPS90 (mean sizes 84.14 μm), MPS60 (mean sizes 61.74 μm), MPS40 (mean sizes 37.39 μm) and MPS20 (mean sizes 22.33 μm) were intragastrically administered with 0.01 mL/g/d WBDF of different particle sizes every day, and NC and CMNC mice were intragastrically administered with 0.9% normal saline for 10 days, twice a day. The experiment was reviewed and approved by the Beijing Vital River Laboratory Animal Management Committee (IACUC No.: P2021029).

### 2.5. Measurement of Constipation Indices

#### 2.5.1. Fecal Moisture Content and Defecation Time of First Dark Stool

The defecation test was performed as follows [27]. On the ninth day of intervention, after intragastric administration, the feces of the mice were collected. The feces were then counted, weighed, and thoroughly dried. The formula used for fecal moisture content calculation was as follows:

(4) Fecal water content (%)=(fecal wet weight−fecal dry weight)/(fecal wet weight)×100 %

Activated carbon, which gives the feces and intestinal contents a black color, is often used as an indicator to describe the state of defecation and gastrointestinal tract in mice [28]. On day 10, all mice were administered an intragastric dose of activated carbon meal solution. The mice were then transferred to individual cages and provided with food and water. The defecation time of the first black stool in each mouse was recorded [29].

#### 2.5.2. Gastric Residual Rate

Mice (*n* = 8) were fasted for 12 h after the end of treatment and then gavaged with 0.2 mL of a nutritive semi-solid paste. After all mice were euthanized, the stomach was then excised for further analysis. The full stomach was weighed, and the weight was recorded. The stomach after removal of gastric contents was used as the net weight of the stomach [30]. The calculation method of residual rate of gastric contents is as follows:

(5) Gastric residual rate (%)=(full stomach weight−net stomach weight) /Semi−solid paste weight×100 %

#### 2.5.3. Intestinal Propulsive Rate

Transit time was estimated, with previous methods serving as guidance [31]. The intestinal propulsive rate specifically was determined by the method reported earlier [32,33]. The entire small intestine of the mice was quickly removed. Total length of the small intestine and the carbon paste propulsion length was measured. The small intestine propulsion rate was calculated.

(6) Intestinal propulsive rate (%)=Carbon paste propulsion length/total intestinal length×100 %

The blood was collected and naturally solidified. Then, it was centrifuged, and the supernatant was collected. Blood and feces were stored at −80 °C.

### 2.6. Biochemical Assays

The serum levels of motilin, gastrin, somatostatin, and acetylcholinesterase activity were determined by use of the commercial ELISA kits (Meimian Industrial Co., Ltd., Yancheng, China).

### 2.7. Fecal Microbiome Profiles by 16S rRNA Gene Sequencing

The gut microbiome was analyzed using 16S rRNA gene sequence amplification analysis. DNA extraction from samples was performed according to the methods described in previous studies [34,35]. After passing the test, the samples continued to be amplified by qPCR. Finally, samples were pair-end sequenced using the Illumina Miseq platform. Illumina sequencing was assisted by E-GENE (Shenzhen, China).

Based on the normalized operational taxonomic unit (OTU) species abundance profile, diversity and relative abundance indices of the OTUs were analyzed. Additionally, species composition was further statistically analyzed at various taxonomic levels through species annotation. The alpha diversity of the samples was evaluated using Shannon, Faiths PD, and Chao1 indexes [36]. The overall structure of the gut microbiome was analyzed using beta diversity through partial least squares discriminant analysis (PLS-DA) [37].

### 2.8. Statistical Analysis

Statistical analysis was conducted using SPSS 24.0 software. Calculated data for each group were expressed as mean ± standard deviation (X ± SD). One-way analysis of variance (ANOVA) was performed for statistical analysis, followed by Duncan’s comparison test to determine significant differences. A *p*-value of <0.05 was considered statistically significant. Microsoft Excel 2019 was used to process data statistics and draw images.

## 3. Results

### 3.1. Physicochemical Properties

#### 3.1.1. Particle Size Distribution

Table 1 shows that different treatments affected the particle size of WBDF differently. The mean particle size was 84.14 μm, 61.74 μm, 37.39 μm, and 22.33 μm. Consistent with previous studies, the particle size was further reduced to 12.2 µm with longer ball milling times [38]. The smallest particle size of WBDF was observed after ball milling, while the biggest particle size underwent treatment by a pulverizer.

#### 3.1.2. FT-IR

**Figure 1:**
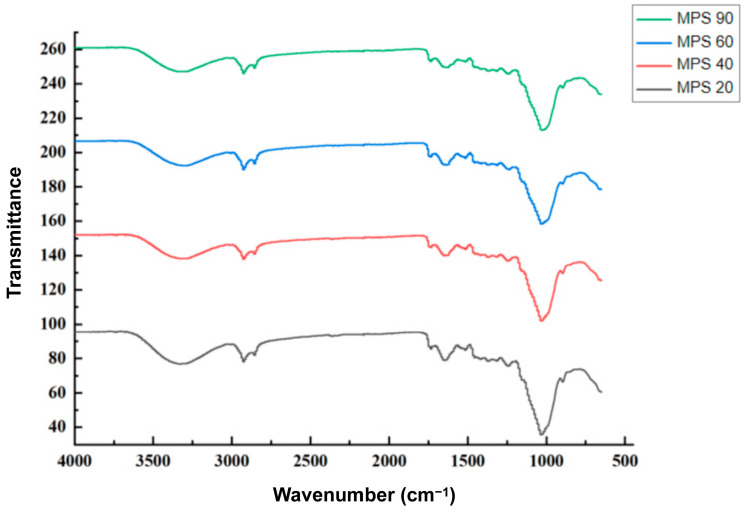
Fourier-transform infrared (FT-IR) spectroscopy of different particle sizes of wheat bran dietary fiber. MPS90: mean particle size of 84.14 μm; MPS60: mean particle size of 61.74 μm; MPS40: mean particle size of 37.39 μm; MPS20: mean particle size of 22.33 μm.

The four different WBDF samples exhibited similar infrared absorption peaks with no new absorption peaks observed (Figure 1). They all had a broad peak at 3423 cm^−1^–3430 cm^−1^ (O-H stretching), a characteristic absorption peak at 2920 cm^−1^ (C-H stretching in carbohydrates), an absorption peak at 1630–1646 cm^−1^ (COO-), and a narrow peak at 1031 cm^−1^ (C-O-C and C-O-H). These absorption peaks indicated that WBDF possessed the typical structure of cellulosic polysaccharides. A higher number of -OH and -COO- typically promoted the dissolution of dietary fibers and increased their water absorption capacity, potentially improving gut health. Carboxyl groups could help form gel-like substances and played an important role in modulating the gut microbiota.

#### 3.1.3. SEM

**Figure 2:**
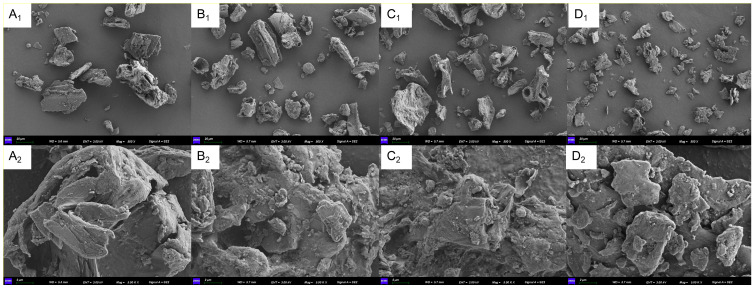
Scanning electron microscope (SEM) images depict different particle sizes of wheat bran dietary fiber. (**A_1_**) MPS90 (500×); (**B_1_**) MPS60 (500×); (**C_1_**) MPS40 (500×); (**D_1_**) MPS20 (500×); (**A_2_**) MPS90 (5000×); (**B_2_**) MPS60 (5000×); (**C_2_**) MPS40 (5000×); (**D_2_**) MPS20 (5000×). MPS90: mean particle size of 84.14 μm; MPS60: mean particle size of 61.74 μm; MPS40: mean particle size of 37.39 μm; MPS20: mean particle size of 22.33 μm.

The microstructural characteristics of DF were related to factors such as the source of the raw material, processing methods, particle size, surface properties, chemical composition, and crystallinity. As shown in Figure 2, the WBDF with different particle sizes exhibits varying microstructural characteristics. At a lower magnification (500×), WBDF with different sizes showed an irregular and rough structure. At a higher magnification (5000×), rougher and more irregular small layers and pores were observed on the surfaces of the MPS60 and MPS40 groups. In contrast, the MPS90 group exhibited large sheet volume and a relatively smooth and flat surface. The above results showed that colloid mill and ultra-high pressure homogenizer treatment had a certain effect on the microstructure of WBDF. The treated samples exhibited more loosely arranged structures and exposed additional functional groups.

#### 3.1.4. WHC, OHC, and SC

**Figure 3:**
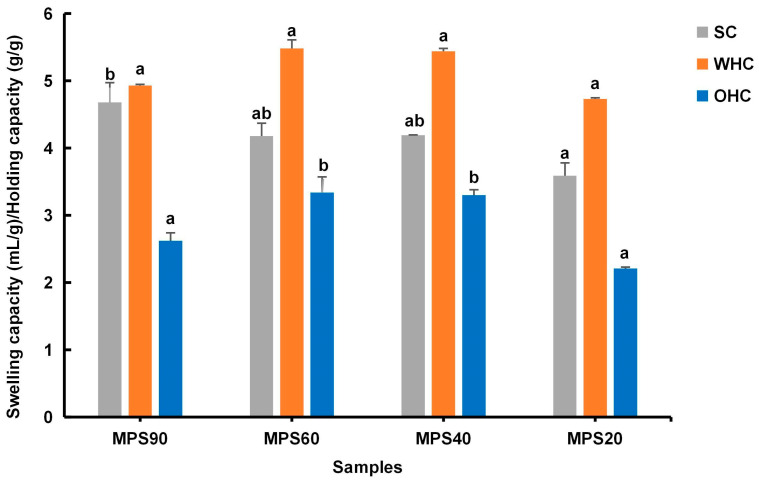
Physicochemical characteristics of different particle sizes of wheat bran dietary fiber. SC, swelling capacity; WHC, water-holding capacity; OHC, oil-holding capability. MPS90: mean particle size of 84.14 μm; MPS60: mean particle size of 61.74 μm; MPS40: mean particle size of 37.39 μm; MPS20: mean particle size of 22.33 μm. Dissimilar lowercase letters signify statistically significant alterations between groups (*p* < 0.05).

The WHC, OHC, and SC of DF were influenced by factors such as particle size, surface properties, chemical composition, temperature, pH, and moisture content. As shown in Figure 3, as particle size decreased, the WHC of WBDF did not change significantly, but there was a trend of rising first and then decreasing. The OHC firstly increased as particle size decreased from 84.14 μm to 37.39 μm, while it decreased once the particle size reached 22.33 μm. SC decreased with particle size reduction. This could be because the grinding process reduces particle size and fractures the fiber matrix and pores, leading to change in the physical properties of the WBDF [39].

### 3.2. Effect of WBDF on Excretion Indices in Constipated Mice

**Figure 4:**
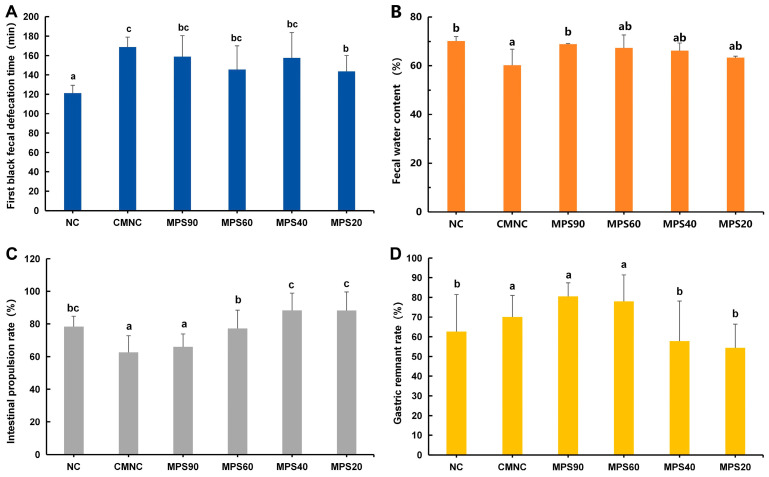
Effects of different particle sizes of wheat bran dietary fiber on defecation function in mice. (**A**) First black fecal defecation time. (**B**) Fecal water content. (**C**) Intestinal propulsion rate. (**D**) Gastric remnant rate. NC, normal control group; CMNC, loperamide induced constipation model group; MPS90, mean particle size of 84.14 μm; MPS60, mean particle size of 61.74 μm; MPS40, mean particle size of 37.39 μm; MPS20, mean particle size of 22.33 μm. Dissimilar lowercase letters signify statistically significant alterations between groups (*p* < 0.05).

To further examine the relieving effect of WBDF with different particle sizes on constipation in mice, changes in fecal moisture content, the defecation time of the first dark stool, gastric residual rate, and intestinal propulsive rate in mice were studied. During the construction of the constipation model, the CMNC group had a sluggish mental state, dull fur, and decreased appetite, and the shape of the feces was rice-like, sharp at both ends, and the color was brown and black. The CMNC group’s defecation time was 39.26% longer than that of the NC group, as seen in Figure 4A, suggesting that the constipation model was successfully constructed. Furthermore, the results showed that WBDF decreased the defecation time by 5.78–14.95% (Figure 4A), with the shortest time observed in the mice administered the MPS20 group (*p* < 0.05). The fecal water content in the different groups of mice is shown in Figure 4B. Compared with the NC group, the fecal water content in the CMNC group was significantly reduced. Fecal water content decreased as the particle size of WBDF decreased. Compared with the CMNC group, the fecal water content in the MPS90 group was significantly increased, while no significant effects were observed between the other three groups.

This study investigated the effects of WBDF with varying particle sizes on the peristaltic function of the gastrointestinal tract in mice. Compared with the NC group (Figure 4C), the small intestinal propulsion rate in the CMNC group was significantly reduced. In contrast, the small intestinal propulsion rate in the MPS60, MPS40, and MPS20 groups increased by 20.32%, 41.21%, and 41.05%, respectively. However, there were no significant differences between the CMNC and MPS90 groups. Additionally, WBDF in the MPS40 and MPS20 groups was shown to considerably lower the stomach residual rate in mice by 19.69% and 30.74%, respectively, according to measurements of the gastric emptying rate (Figure 4D). These results suggested that the particle size of WBDF influenced gastrointestinal peristalsis in constipated mice.

### 3.3. Effect of WBDF on Contents of GI Hormones in Mice

**Figure 5:**
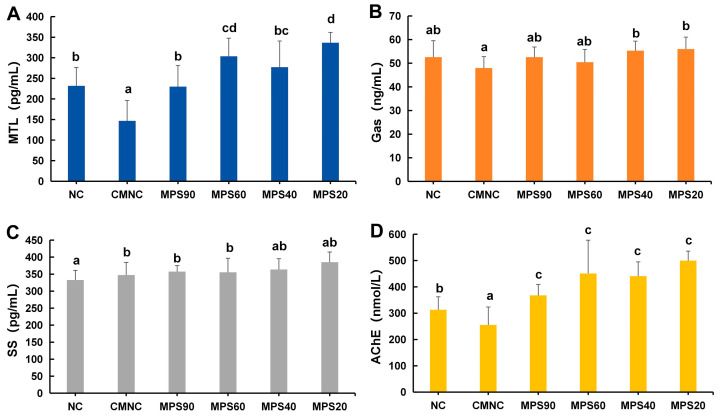
Effects of different particle sizes of wheat bran dietary fiber on the contents of (**A**) motilin (MTL), (**B**) gastrin (Gas), (**C**) somatostatin (SS), and (**D**) acetylcholinesterase (AChE). NC, normal control group; CMNC, loperamide induced constipation model group; MPS90, mean particle size of 84.14 μm; MPS60, mean particle size of 61.74 μm; MPS40, mean particle size of 37.39 μm; MPS20, mean particle size of 22.33 μm. Dissimilar lowercase letters signify statistically significant alterations between groups (*p* < 0.05).

Figure 5 shown the changes in serum motilin (MTL), gastrin (Gas), somatostatin (SS), and acetylcholinesterase (AChE) levels in different groups of mice. Serum analysis revealed that, compared to the NC group, the MTL content in the CMNC group decreased to 146.68 ± 49.62 pg/mL (Figure 5A). Between the treatment groups, MTL levels were 56.86–129.46% higher than in the CMNC group, with the highest content observed in mice administered the MPS20 group (*p* < 0.05). The Gas content in the CMNC group decreased, but it was not significantly different from the NC group. In contrast, the MPS40 and MPS20 groups exhibited a notable increase in Gas levels compared to the CMNC group (Figure 5B). The levels of SS did not show significant changes between the treatment groups, but there were significant differences between NC and CMNC, MPS90, and MPS60 (Figure 5C). Additionally, AChE content decreased from 312.86 mmol/L in the NC group to 255.36 mmol/L in the CMNC group, with a significant difference observed between the two groups (Figure 5D). In the treatment groups, AChE levels increased by 51.12–105.29% compared to the CMNC group, with the highest content observed in the mice administered the MPS20 group (*p* < 0.05), while no statistical differences were found among the treatment groups. The results suggested that WBDF treatment could alleviate constipation symptoms in loperamide-treated mice, and the smaller the particle size, the better the effect.

### 3.4. Effect of WBDF with Different Particle Sizes on Gut Microbiota

**Figure 6:**
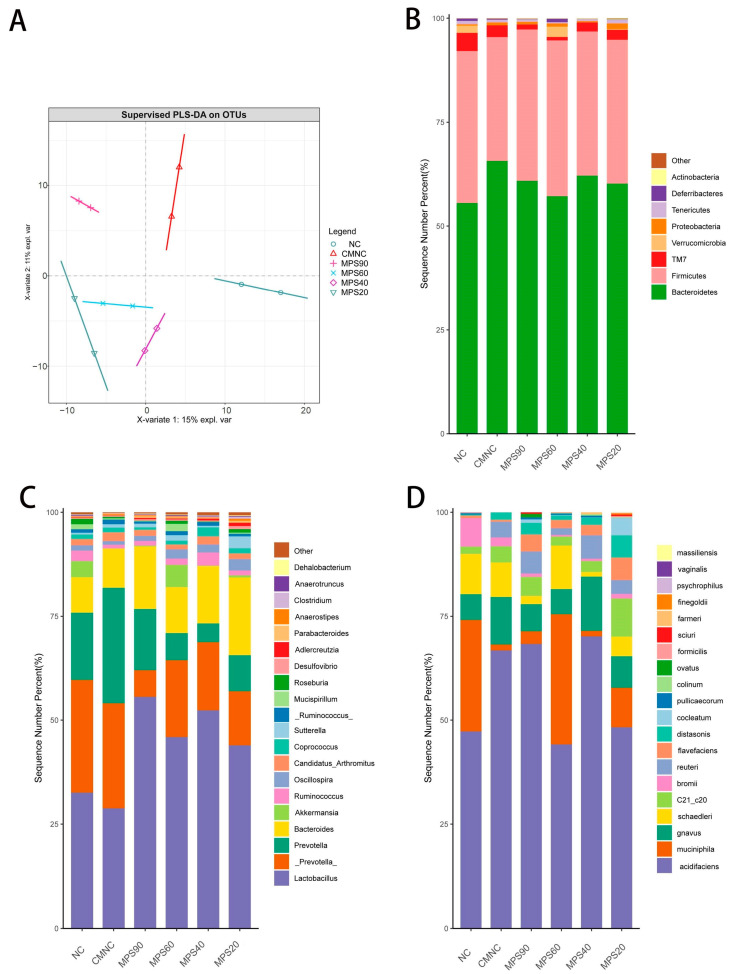
(**A**) PLS-DA of gut microbiota at the OTU level. Comparing the composition of the different gastrointestinal tract microbiome at (**B**) the phylum level, (**C**) the genus level, (**D**) and the species level in all groups of mice (*n* = 8). PLS-DA: partial least squares discriminant analysis; OTU: operating taxonomic unit, NC, normal control group; CMNC, loperamide induced constipation model group; MPS90, mean particle size of 84.14 μm; MPS60, mean particle size of 61.74 μm; MPS40, mean particle size of 37.39 μm; MPS20, mean particle size of 22.33 μm.

As shown in Table 2, all WBDF of different particle sizes could increase the Shannon index and Chao1 index, demonstrating that richness and diversity of gut microbiota were able to improve. The microbiota makeup of the several groups varied significantly, as shown by the PLS-DA analysis (Figure 6A).

At the Phylum level (Figure 6B), the bar charts illustrated that the majority of the sequences were classified under Firmicutes and Bacteroidetes. Relative abundance of Firmicutes in the CMNC group (29.80%) showed a decreasing trend. In contrast, treated by WBDF, the abundance of Firmicutes increased to different degrees. On the other hand, the abundance of Bacteroidetes was significantly increased in constipated mice, while it significantly decreased by intaking WBDF. And the MPS60 group had the most obvious effect, reducing 57.15% of the Bacteroides amount.

At the genus level (Figure 6C), the sequences identified included *Lactobacillus*, *Prevotella*, *Bacteroides*, *Akkermansia*, and *Lachnospiraceae*, with *Lactobacillus* and *Prevotella* being the most abundant. DF of varying particle sizes significantly enhanced the abundance of *Lactobacillus* in constipated mice, especially in the MPS90 (56.45%) and the MPS40 groups (52.52%). Compared to the NC group, the abundance of *Akkermansia* in the CMNC group decreased by 98.39%, while the levels of *Akkermansia* in the MPS60 group significantly increased. *Prevotella* grew vigorously in a pro-inflammatory environment and even promoted the development of inflammation. Following WBDF treatment, the relative abundance of *Prevotella* was also markedly lower; in particular, the relative abundance in the MPS40 group was 83.55% lower than that in CMNC group. Interestingly, compared to the control group, its proportion was also lower. This may be due to the presence of bioactive compounds in WBDF, such as polysaccharides, phenolic compounds, or antimicrobial peptides that inhibit the growth of Prevotella, or because WBDF promotes the growth of other microbes, affecting its survival. However, the specific mechanism behind this phenomenon requires further investigation.

At the species level (Figure 6D), *Acidifaciens*, *Muciniphila*, *Gnavus*, and *Schaedleri* were dominant in the intestinal microbiota of each group. Compared to the NC group, the abundance of *Acidifaciens* and *Gnavus* increased in the CMNC group, while the abundance of *Muciniphila* did not increase significantly in all treatments. After DF treatment, particularly in the MPS60 and MPS20 groups, the abundance of *Acidifaciens* and *Gnavus* decreased significantly, while the abundance of *Muciniphila* increased significantly. Additionally, the abundance of *Schaedleri* was higher in the MPS60 group compared to the CMNC group.

WBDF was beneficial for the growth of *Lactobacillus* and *Akkermansia*, and it could maintain an acidic environment and health in the intestines.

## 4. Discussion

In this study, the structural characteristics of WBDF with different particle sizes were investigated, and its effects on constipation were evaluated. The chemical functional group structure of WBDF remained unchanged after treatment. The FT-IR spectrum (Figure 1) showed that all four samples contained uronic acid and possessed a pyranose ring structure, exhibiting characteristic peaks of fiber polysaccharides [40,41,42]. In addition, SEM results (Figure 2) showed that both pulverizer and ball milling treatments decreased the particle size, and WBDF microstructure became looser and more porous, similar to previous studies [43]. These changes suggest an increase in potential enzymatic hydrolysis sites and an improvement in functional properties [44].

The WHC and OHC of WBDF showed a trend of initially increasing and then decreasing. This may be due to the fact that, as the particle size decreases, the hydrophilic groups are more exposed, and some IDF is converted into SDF, which increases the viscosity of the system, leading to greater water absorption and swelling, thereby enhancing moisture binding. Furthermore, the smaller particle size of WBDF increases the surface area available for oil absorption, and the fiber structure composed of fine particle samples is more loosely arranged with more capillary pores, allowing for greater oil penetration [45]. However, the holding capacity became lower when the particle size was below a particular value. This could be because smaller particles have a greater contact area, yet their individual binding capacities are reduced [46]. A similar conclusion has been drawn in studies showing that small particles (85 to 150 μm) of wheat bran have lower WHC compared to large particles (150 to 400 μm) of wheat bran [47].

Clinical signs of constipation include prolonged gastrointestinal defecation time, tension during feces, and incomplete defecation [1]. Initial research revealed that WBDF promoted fecal output, decreased the defecation time, and increased the fecal water content (Figure 4). This could be because an appropriate amount of DF treatment can soften hard feces, increases the rate of fecal excretion, makes feces easier to pass, and prevents their accumulation in the intestine [48]. Additionally, intestinal peristalsis is the main driving force for the movement of intestinal contents [49]. As the particle size decreased, the intestinal propulsion rate of the mice gradually increased (Figure 4C). This could be attributed to the reduction in both the viscosity and frictional resistance of WBDF as the particle size decreases, which consequently leads to an increase in the intestinal propulsion rate [15]. A lower gastric residual rate in mice indicates a faster gastric emptying rate, suggesting enhanced gastric motility. The ability of WBDF with smaller particle size significantly increased the intestinal propulsion rate and decreased the gastric residual rate in mice. This indicates that the addition of DF with small-sized particles to the diet of healthy mice can promote intestinal peristalsis and enhance gut function.

Gastrointestinal hormones serve as constipation indices and play crucial roles in regulating the secretion of digestive glands and gastrointestinal motility [32]. Previous studies have indicated that gastrointestinal motility can be improved by modulating gastrointestinal hormone secretion [50]. MTL and Gas enhances pyloric sphincter contraction due to their beneficial effects on smooth muscle contraction and gastrointestinal motility [51]. On the other hand, SS acts as an inhibitory peptide that suppresses gastrointestinal hormone secretion. As loperamide hydrochloride achieves its antidiarrheal effect mainly by inhibiting smooth muscle contraction, it significantly affects the levels of MTL and AChE, both of which regulate smooth muscle contraction. DF has the potential to regulate the levels of intestinal excitatory neurotransmitter parameters in constipated mice, as suggested by Liu et al. [52]. Figure 5 showed that WBDF increased the levels of MTL, Gas, and AChE in mouse serum. Significant variations in the levels of MTL and AChE were observed when comparing the WBDF treatment groups with the CMNC group, with the highest levels of both hormones observed in the MPS20 group, which is consistent with previous studies [27,53]. Yang et al. reported that the Gas levels in CW_1_._8_ (small particle size) mice were significantly higher than those in CW_0_._6_ (large particle size) mice [15]. Similarly, our study demonstrated that when the particle size of WBDF was below 40 μm, the Gas content significantly increased. These results prove that WBDF can affect constipation caused by loperamide hydrochloride, with the smaller-particle-size group showing stronger effects than the larger-particle-size group.

The development of constipation is significantly influenced by the gut microbiota [36]. Consistent with previous research, treated by WBDF, the abundance of Firmicutes increased to different degrees [54]. *Lactobacillus* accumulates saturated long-chain fatty acids, which encourage colonic muscle contraction and increase the frequency of bowel movements [55]. The MPS90 group (56.45%) and the MPS40 group (52.52%) significantly enhanced *Lactobacillus* abundance, improving the condition of constipated mice (Figure 6C). This result is similar to the findings of Tang’s study, where SC (seaweed cellulose) intervention mitigated the negative effects of HFSD (high-fat sugar diet), preventing a decrease in the relative abundance of *Lactobacillus* [56]. Additionally, treatment with WBDF notably increased the levels of *Akkermansia* in constipated mice. Previous studies have indicated that *Akkermansia* plays a key role in maintaining intestinal mucosal integrity and modulating immune responses. The mucus layer, primarily composed of mucins, usually covers intestinal epithelial cells (IECs), providing protection against microbial invasion and serving as a source of nutrients for microbial growth [57]. Dietary fiber increases the secretion of mucins from the intestinal mucosa into the gut lumen, providing more substrates for the growth and proliferation of symbiotic bacteria. This enhances the levels of probiotics, thickens the intestinal epithelium, and improves the integrity of the mucus layer, thereby promoting intestinal homeostasis [58]. Following WBDF treatment, the relative abundance of *Prevotella* was also markedly lower, especially in the MPS40 group. The results align with the findings of Liu et al., who demonstrated that Zengye decoction could reduce the level of harmful bacteria, such as *Desulfovibrio*, *Ruminococcus*, *Prevotella*, and *Dorea* [59].

## 5. Conclusions

In summary, the WBDF treated by the ultra-high-pressure homogenizer (MPS40) had a looser and more porous microstructure and better WHC and OHC, effectively regulated gastrointestinal function, and improved constipation symptoms in mice. WBDF samples with different particle sizes regulated constipation by increasing beneficial bacteria while decreasing *Prevotella* and *Bacteroides*. This study provides a rationale for promoting intestinal health by utilizing WBDF, enhances the understanding of food health factors, provides a follow-up application direction, and supports the advancement of intestinal health food production. However, further research should be conducted on the metabolic regulation pathways in the intestines of mice to alleviate constipation symptoms, as well as the effects of WBDF from wheat bran with more various particle sizes and their impact on the human body.

## Figures and Tables

**Table 1:** Particle size of wheat bran dietary fiber treated by different methods.

Method	Disintegrator	Colloid Mill	Ultra-High Pressure Homogenizer	Ball Mill
Median particle size	101.72 ± 0.99 ^a^	72.80 ± 2.53 ^b^	41.98 ± 3.34 ^c^	20.55 ± 1.34 ^d^
Modal particle size	119.46 ± 14.53 ^a^	115.54 ± 5.79 ^a^	53.34 ± 5.78 ^b^	12.53 ± 5.63 ^b^
Mean particle size	84.14 ± 2.29 ^a^	61.74 ± 1.61 ^b^	37.39 ± 2.66 ^c^	22.33 ± 1.99 ^d^
Samples	MPS90	MPS60	MPS40	MPS20

MPS90: mean particle size of 84.14 μm; MPS60: mean particle size of 61.74 μm; MPS40: mean particle size of 37.39 μm; MPS20: mean particle size of 22.33 μm. Dissimilar lowercase letters signify statistically significant alterations between methods (*p* < 0.05).

**Table 2:** Effect of different particle sizes wheat bran dietary fiber of microbial α diversity of fecal intestinal microorganisms in mice with constipation.

Group	NC	CMNC	MPS90	MPS60	MPS40	MPS20
Chao1	260.88 ± 2.53 ^ab^	181.04 ± 63.70 ^a^	315.65 ± 8.63 ^b^	352.11 ± 10.40 ^b^	288.78 ± 5.26 ^b^	327.70 ± 104.23 ^b^
Faith’s pd	13.66 ± 2.46 ^a^	13.94 ± 7.03 ^a^	17.57 ± 2.33 ^a^	17.16 ± 0.63 ^a^	15.97 ± 0.40 ^a^	16.68 ± 2.97 ^a^
Shannon	5.99 ± 0.31 ^b^	5.30 ± 0.26 ^a^	6.08 ± 0.20 ^b^	6.18 ± 0.05 ^b^	6.12 ± 0.22 ^b^	6.35 ± 0.59 ^b^

NC, normal control group; CMNC, loperamide induced constipation model group; MPS90, mean particle size of 84.14 μm; MPS60, mean particle size of 61.74 μm; MPS40, mean particle size of 37.39 μm; MPS20, mean particle size of 22.33 μm. Dissimilar lowercase letters signify statistically significant alterations between groups (*p* < 0.05).

## Data Availability

The original contributions presented in this study are included in the article. Further inquiries can be directed to the corresponding authors.

## Abbreviations

The following abbreviations are used in this manuscript:

AChE	acetylcholinesterase
DF	dietary fiber
FT-IR	Fourier-transform infrared
Gas	gastrin
IDF	insoluble dietary fiber
MPS	mean particle size
MTL	motilin
OHC	oil-holding capacity
OTU	operational taxonomic unit
SC	swelling capacity
SDF	soluble dietary fiber
SEM	scanning electron microscope
SS	somatostatin
WBDF	wheat bran dietary fiber
WHC	water-holding capacity
